# Supplementary material for: ExposoGraph: An Interactive Platform for Carcinogen Bioactivation and Detoxification Pathway Visualization
Source: Med Oncol. 2026 May 12;43(6):187. doi: 10.1007/s12032-026-03297-4 (PMC13179218; doi:10.1007/s12032-026-03297-4)

Figure S1. Baseline versus the CYP1A1*2C / GSTM1-null high-risk genotype.


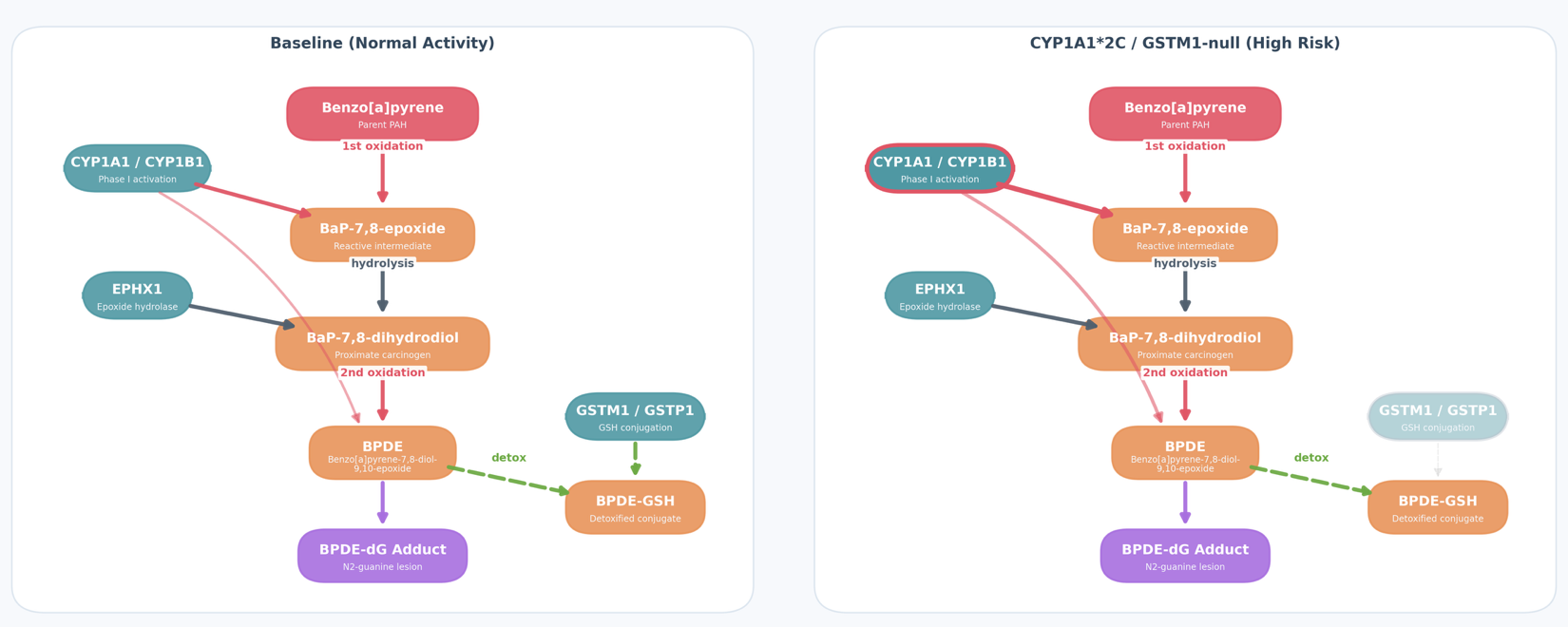


Figure S2. Baseline versus the reduced detoxification.


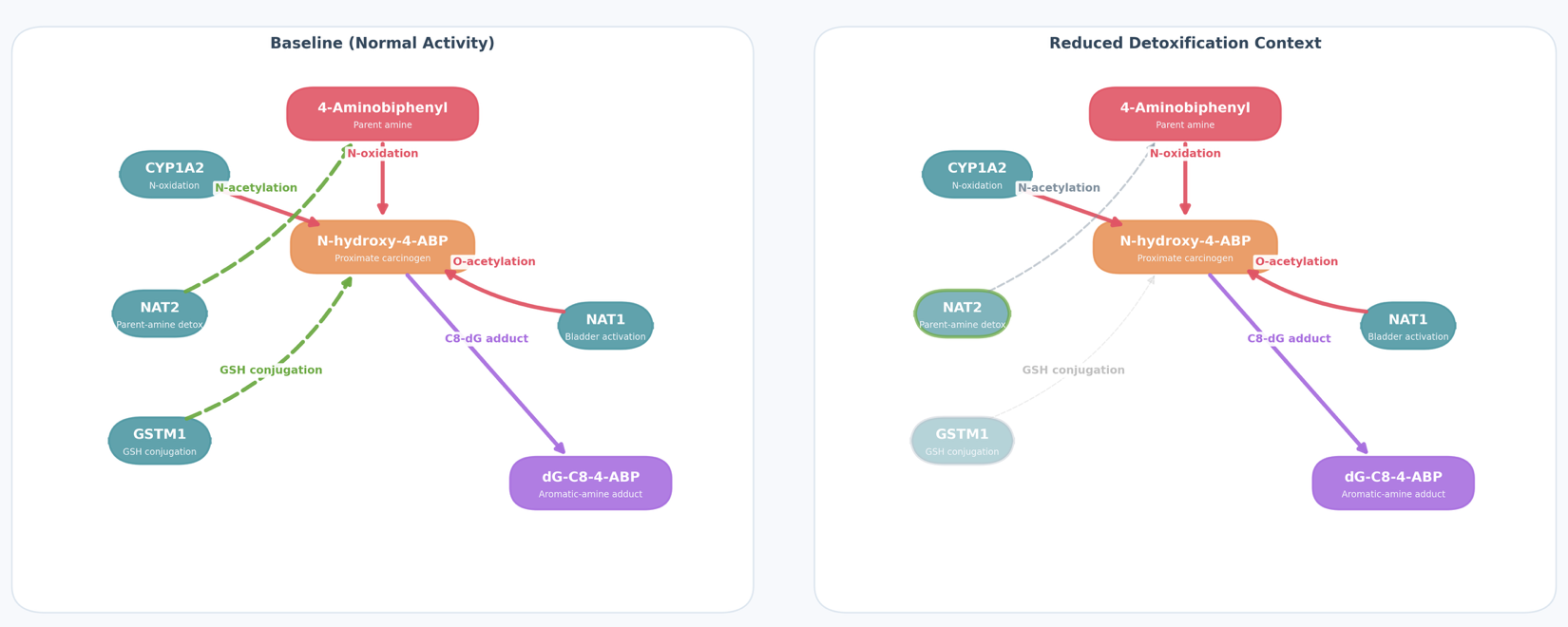

Supplement: Supplementary file 1 — Supplementary Material 1 [file 12032_2026_3297_MOESM1_ESM.docx]
